# Care Ethics Management and Redesign Organization in the New Normal

**DOI:** 10.3389/fpsyg.2021.747617

**Published:** 2021-12-15

**Authors:** Silvio Carlo Ripamonti, Laura Galuppo, Sara Petrilli, Sharon Dentali, Riccardo Giorgio Zuffo

**Affiliations:** Catholic University of the Sacred Heart, Milan, Italy

**Keywords:** human resources, new normal, COVID-19 pandemic, care ethic management, qualitative studies

## Abstract

The pandemic period has placed the organizations in a state of great tension. It has generated a situation of confusion, lack of rules, and production-related criticalities that have called into question the very existence of many productive realities. This article aims to highlight the dimensions of care and ethics put in place by HR managers in COVID-19. The objective that animated the authors have focused on the HRM level of medium and large companies in Italy to highlight the protective actions toward people and the organization in the period COVID 19, highlighting what were the ethical values and actions of care put in place. In this article, we wanted to give voice to managers (*N* = 45, including 21 women and 24 men, aged between 40 and 55 years old) who had management tasks in their organizations by asking them to tell us how they dealt with the challenges imposed by the emergency. In the research, we start from a way of understanding workplaces understood as a “process of ongoing social relationship” within which the HR function is dedicated to the care of the quality of relationships. HR managers have to manage a complex role of mediating between the interests of people and employers by trying to find good mediations.

## Introduction

The pandemic period has placed the organizations in a state of great tension. It has generated a situation of confusion, lack of rules, and production-related criticalities that have called into question the very existence of many productive realities. These difficulties were compounded by sometimes contradictory and unclear government communication. As a recent study (Salem et al., 2021) shows, in times of crisis, government support and clear communication are critical ingredients in dealing with an emergency such as a pandemic. In the Italian context, many managers of large companies reported a lack of clear stance from national and regional authorities. Organizations had to position themselves, especially in the first phase of the pandemic, by deciding on priorities and where to devote more resources and efforts.

The changes introduced by the pandemic have radically changed many of the anchors that qualified the job place. The most crucial difference is undoubtedly related to the worker-workplace relationship. The pandemic has forced everyone to work at home, and this trend will continue even after the emergency is over. The pandemic has forced everyone to work at home, and this trend will continue even after the crisis has ended. This change requires a radical rethinking of the relationship with the workplace since a fundamental link between workers and workplaces has been broken, calling into question people’s place attachments to their workplaces. In the literature, the construct of place attachment is related to the possibility of implementing prosocial behavior and pro-environmental behavior (Ramkissoon, 2020a,b). This forced management to rethink what kind of support they could put in place to ensure that their organization continued to function as effectively as possible.

Companies, therefore, have introduced new ways of working, increasing the use of artificial intelligence (Vergine et al., 2019), rethinking a new reorganization of workspaces (Crowley et al., 2020) and increasingly using remote working (Green et al., 2020; Sadovyy et al., 2021).

This article highlights how managers gave meaning and embellished “care” during the spread of the COVID-19 pandemic. Since work is understood as a process of ongoing social relationships, the study attempts to contribute to the development of research on the “ethics of care” and caring relationship at work, which is still at the beginning (Islam, 2013; Fassauer, 2019). HR managers are particularly suitable for investigating care in work organizations, mediating between the firm’s interests and people’s wellbeing. Many research studies have testified to recurring difficulties reconciling these two stakes (Azambuja and Islam, 2019). Polarized situations are often created where conflicts arise, and managers are involved in contradictory requests and dilemmas, difficult to reconcile. Among the other roles, HR managers are the ones who are more related to the difficulty of balancing “care” and “control” in the workplace. Several authors have investigated what HR roles are more associated with care (see, for instance, Ulrich, 1996). The emotional labor related to caring (O’Brien and Linehan, 2014), to date, there is still a lack of research about the management’s subjective view of care and the perceived dilemmas and challenges implied in their caring efforts in different contexts and situations. The COVID-19 spread has highlighted the need for people care in many contexts and has pushed managers even further toward this direction by raising new awareness about this topic. Our conviction is that the ways managers handled the emergency might have consequences in the medium and long term.

In this article, we wanted to investigate how HR managers dealt with the challenges imposed by the COVID-19 emergency and how they related such actions to the concept of “care.” This investigation could help highlight challenges, contradictions, opportunities, and threats for the future (Vergine et al., 2019).

In the study, researchers interviewed 45 HRM asking questions about how work changed during the pandemic. The researchers also investigated how workers were supported during the emergency. In their narratives, managers made explicit the “implicit theories” that guided them to caring actions in times of crisis.

Managers’ narratives have been treated through an interpretive and hermeneutic approach where accounts rely on subtlety and sometimes taken-for-granted ideas about the nature of workplace relationships and responsibilities with others (Scaratti et al., 2014; Filstad et al., 2019; Cunliffe and Ivaldi, 2021). We, therefore, collected multiple interpretations and values around “caring” issues that helped compose a multifaceted and nuanced picture of HRM efforts in the pandemic.

One critical theme emerged. It is related to different objects of “caring:” work, people, and organization. These three objects seemed closely associated with how HMR perceived their role in their organization and prioritized their efforts during the pandemic. Within each of the three objects, managers, therefore, highlighted further challenges due to several perceived contradictions, which recall the well-known HR dilemma between “caring” vs. “control;” business vs. people “survival.” We hypothesize that such contradictions and the ways managers face them might significantly impact the HR role in the future. The report, therefore, ends up with some reflections and indications for further investigation in the field.

## The HRM “Ethics of Care”

In the present report, we ground our reflections in the ethics of care approach (Gilligan, 1982; Fassauer, 2019; Tomkins and Bristow, 2021). This highlights as a framework for guiding and evaluating action and interaction, especially between those who wield power (e.g., managers) and those in need or trouble. A care ethical path to management practices focuses on how an action or intervention will affect particular people in particular circumstances ahead of the abstract criterion of whether it is right or wrong or the instrumental criterion of whether “it works.” Caring is about considering what “matters,” rather than what “works” in a given situation.

This approach focuses on what Gherardi and Rodeschini (2016) calls the “doing” of care, a local focus on the social environment in which situated actions take place, rather than a universal and normative approach based on abstract claims. Therefore, the “ethics of care” is embedded (Cunliffe and Ivaldi, 2021): behaviors that are embedded in managers’ work practices and that express how ethical values are acted out in everyday choices.

As a final aspect, the ethics of care approach does not assume managers to be agents who autonomously and rationally decide their course of actions. Still, it considers management as a relational activity, organized around the possibility of being responsive to others’ voices and being influenced by the diversity of desires and interests at play (Gilligan, 1995). In this sense, management “caring” might be open to tensions, contradictions, and dilemmas that must be reflexively considered and handled.

The ethics of care perspective helped us investigate how managers addressed the COVID-19 crisis and the implicit assumptions that moved them. The supportive actions that were put in place in the pandemic expressed managers’ ethical values and orientations and how these helped them handle emerging contradictions. From these assumptions, managers organized their agenda, everyday activities, and how they accounted for them.

## Sample and Methodology

The researchers interviewed 45 managers, including 21 women and 24 men. The managers interviewed are all university graduates, aged between 40 and 55 years old. The sectors they belonged to were manufacturing, IT, and services. Public companies were not included in the sample. The following diagram details the type of organizations involved in our Italian context (Table 1):

**TABLE 1:** The type of organizations involved.

	Textile industry	Chemical industry	Industrial equipment	Energy industry	Telecommunication	Metal working	Total
First level managers	5	4	5	6	3	2	
Second level managers	4	3	4	5	2	2	
	9	7	9	11	5	4	45

The respondents were given a narrative interview outline that primarily touched on the following points:

• Effects of COVID on HR management policies.
• Proposed projects to support people.
• Management priorities over the next 12 months.
• Reconfiguration of how we work with other functions.

The interviews were recorded and disgorged, and we obtained a 380-page corpus, that is. The interview analysis methodology was guided by content analysis (Braun and Clarke, 2006). The researchers independently coded the data corpus. After this step, the study team activated a process of calibrating the codes associated with each. The identified codes were then aggregated into clusters of themes that are discussed in this article.

## Results

**FIGURE 1:**
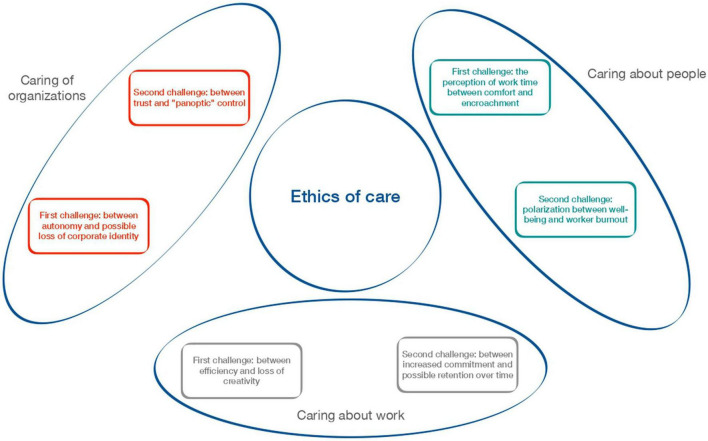
Ethics of care.

The results were organized by identifying some core themes (as represented in Figure 1); all the references to the concept of “caring” were grouped into three different categories, representing three different levels/objects of care: people, work, and organization.

We will now describe the results in detail.

## Caring About People

In this first category are the narratives of managers who, in the pandemic period and in the reorganization that follows, were focused on safeguarding the possibility that people could work in an acceptable state of well-being (Raya and Panneerselvam, 2013). The issues governing their managerial activity centered on helping people contain the time they worked remotely, modulating the commitment over time, and providing support to have a sustainable life-work balance (Koinig and Diehl, 2021). In these narratives, two main challenges emerged.

The first was linked to the encroachment of work into private life, and the second was linked to the balance that people had to build again in a work mode played less and less in presence.

### First Challenge: The Perception of Work Time Between Comfort and Encroachment

The reconfiguration of workers’ time led to a strong sense of ambivalence in managers’ accounts. On the one hand, there was the perception of a great advantage of remote working because it favored the management of work in workspaces that were considered comfortable, close to one’s home, in an environment that protected the worker from the fatigue of commuting. From home, there were all the comforts needed to make the worker feel comfortable. In this way, managers could devote a lot of attention to sharing with their collaborators, thus strengthening their relationship:

*”The great thing about remote working is that it doesn’t force you to clock out, and it increases the employee-manager relationship. One of the phenomena is that this way of working is increasing the quality of performance. Now people don’t have to come to the office but only to share what they need to share with their managers.”*

HRMS also reported another aspect of remote-working. In their accounts, workers were often identified using adjectives that denoted the fatigue in their lives’ immanence of work. People told of “encroachments” of the work that had slowly taken over all the private space of the worker. Every area of life was conquered by online meetings and by cell phones, portable PCs that made managers and employees constantly available.

*”Management must put in place strategies to protect employees’ spaces from containing the phenomena of stress and depersonalization related to the perception of “occupation of one’s home.”*

### Second Challenge: Polarization Between Well-Being and Worker Burnout

HRM described the corporate population often using the term “polarization” between profiles used to self-determine their work and those who found it very difficult to organize themselves without external guidance (Rodríguez-Cifuentes et al., 2020). The firm’s population was described as divided between people who, in essence, were already accustomed to working with significant autonomy and profiles for whom remote operations represented a destabilizing novelty.

*”In some people, there is a slowdown from a responsiveness standpoint. They probably suffer from not being in a constant physical and relationship context with colleagues, and they are slowed down intellectually. Others have become very concrete and high performers because they work in a more relaxed context for them, and it depletes their capacity.”*

Managerial actions were about supporting people by strengthening their ability to manage the reconfigured work relationship. In this category of narratives, the interviewees proposed actions ranging from coaching to rethink the relationship between oneself and one’s work, training centered on time management, and offering psychological support to support individuals in reviewing their relationship with work. The level of managerial action was the individual, and the possibility of affecting the broader class of the group or organization did not seem to be represented.

## Caring About Work

In this category, there are narratives of managers who assumed that the pandemic changes altered the very nature of work. Here again, two main challenges emerged.

### First Challenge: Between Efficiency and Loss of Creativity

The polarity efficiency vs. creativity expresses a considerable challenge that impacted on HRMs’ concerns. Interviewees described remote work with adjectives that denoted the greater concentration and precision provided by working without being disturbed by office dynamics. Working at the times and in the most pleasant spaces helped have higher individual performances. However, all HRM reported that remote working made it much more complex and sterile where it was necessary to achieve creative outcomes and generate new products or ideas.

*”In this hybrid operation, we’ve lost a few pieces in terms of coordination. If you have one part of the team in the office, you communicate more with that part without worrying about absentees. Fully virtual meetings also work well in terms of efficiency man on in terms of creativity. On the other hand, if meetings are done in a hybrid way, they don’t work well, and they don’t work that well.”*

## First Challenge: Between Increased Commitment and Possible Retention Over Time

HRM in the interviews reported that they had seen an increased workers’ engagement with working remotely. Each seemed to be committed in a real battle to cope with the demands of the business, the household tasks that were “closer” remotely. Such commitment appeared self-imposed to show off to bosses who were not as close as before. In the explosion phase of the pandemic, this attitude of strong engagement was also functional in putting more resources into play to help the work and workers’ productivity stay “afloat.” However, managers’ questions started to be: “how long can this phase of increased engagement last?” In a long time, productivity could be lowered and work depowered by such a long-lasting resource-consuming effort.

*”The emergency has led to the company not taking charge of work-at-home situations, but that can’t last. You have to regulate even from the social point of view to how I can work from home. There are personal situations at home that are very complex to live with. The extra performance and reactive to a new working condition.”*

As can be noted, HR managers in this category perceived themselves as “guarantors of the quality of work itself.” thus trying to re-create the conditions for the work to get done. The unit of analysis of their action was the work, like a time/space system resulting from the joint effort of several people and needing to be coordinated in a new way for safeguarding its productivity and sustainability.

## Caring of Organizations

In this third category, the caring efforts concerned the organization as a “complex social subject.” The reconfigured and fragmented work, the acceleration of new technologies, and the latest psychological contract between people and the firm called the survival of organizations into question. The most pressing issues on this level were the possible end of the trust between person and organization and the extreme stress on corporate identity/culture.

Two main challenges were here posited:

### First Challenge: Between Autonomy and Possible Loss of Corporate Identity

This theme referred to the possible loss of moments of exchange between employees that are the foundation of any organizational culture/commitment. Informal interactions between people, the lack of coffee machine work, preambles at meetings, and follow-up comments were described as the forge of the “cultural backbone” of the organization. The pandemic period and the following phase confirmed that organizations would continue to provide a good deal of remote white-collar work. The interviews highlighted this trend as a possible source of risks related to the possibility of transmitting and reinforcing organizational cultures.

*”Companies there are exogenous networks from the organizational structure. Connections and exchanges are created that are the organizational backbone. Remote working will lose that dimension, which comes from the coffee machine and from lunches together.”*

### Second Challenge: Between Trust and “Panoptic” Control

Finally, the theme of control emerged strongly; the unstructured work put managers at a dangerous crossroads. On the one hand, the new reconfigured work, managed by distance, imposed an evident change in personnel management: from control in the presence to delegation and trust. In many cases, this new perspective gave rise to a contrary reactive movement that raised remote surveillance of people (Yaghi et al., 2020). Here then appeared new forms of control as the monitoring of bytes downloaded from pc of the collaborators, monitoring their presence on the business networks that made it possible, the telephone used during working hours to control more than to exchange information.

*”On the company network, when one is connected, you see the little green light, and then as a boss, I am comfortable. I also asked if you could track bytes downloaded during working hours. So, I have presence and productivity under control!”*

In this third category, thus, managers found themselves taking care of the organizational plan, its identity and values, and its survival. The unit of action here was the “organizing” processes, and managers played the roles of controllers, socialization facilitators alternatively, and coaches trusting in their collaborators more than before.

## Conclusion

The three “clusters” of care highlighted in the interviews showed how human resource managers had difficulty containing the situation’s complexity in the pandemic and post-pandemic context.

The challenge of keeping work, people, and organizations together is an ongoing one and is based on three forces which we will list below. The job of managers is expressed primarily in their ability to protect the interconnections among these three levels. The most challenging HR managers interviewed was specifically this ability to “take care” of the interactions between the three levels, work organization and people (Giancaspro et al., 2021). Extreme difficulty in holding these divergent goals together emerged in the interviews. We explain this difficulty by hypothesizing three perhaps that generate this difficulty.

### First Force

Pressure in turbulent contexts. The perception we have had is that the pressure exerted on managers pushes them to some concrete, extremely simplifying style of thinking that does not allow them to realize the complexity of organizational life (Yaghi et al., 2020). The force given by corporate life leads to focus attention on just one prevalent theme of the three identified. This tension can make it difficult for managers to safeguard the needs of workers, starting with the possibility of creating a balanced environment where the well-being of workers can be protected. The concept of the healthy leader in our study sample was hardly made present in everyday managerial action. Therefore, some authors’ calls to interpret the healthy manager’s role (Raya and Panneerselvam, 2013; Koinig and Diehl, 2021) seem to remain as good wishes that have not been fully realized in organizational practice.

### Second Force

The role of professional sub-cultures. The sectorial culture that develops in organizations does not help build a multi-perspective view that considers the complexity of reality. Professional affiliations lead people to focus their gaze on the issue that identifies their professionalism. In this study, we interviewed all HR managers. However, we know that within the HR world, there are professional sub-cultures to which people belong that influence the way they work. To identify the significant professional sub-cultures in the HR world, we can refer to the well-known Ulrich (1996) scheme that identifies four principal roles: Employ Champion, Administrator, Changing manager, HRBP. We noticed that HR people are strongly conditioned by the professional culture linked to their role in the interviews. Those in planning and budgeting are more focused on the issue of organizational survival and labor costs. HRBPs who are much closer to line managers highlighted the importance of quality of work issues. HRs focused on the People Advocate role expressed more attention to people retention and resilience. Each of these three professional roles, in other words, interpreted the topic of ethics from their professional perspective.

### Third Force

The role of organizational culture. Organizational cultures impose priorities and generate sensitivities in managers. Thus, some cultures are more focused on costs and leave people’s well-being in the background.

Technocratic cultures are susceptible to innovation and quality of work. Organizations that are mainly focused on the well-being of their employees tend, instead, to devote resources and time to increase the quality of life within them (Di Fabio et al., 2016).

The intertwining of these dimensions generates different forms of embedded ethics in organizational practices (Mihalache and Volberda, 2021). At a paradigm shift, the ethics of care for managers is of primary interest to put the worker at center stage, safeguarding competitiveness and the survival of the dignity of work and organizational sustainability. It is necessary to support the development of multi-perspective managerial thinking that can deal with the complexity of reality rather than the simplifying revision that emphasizes the poles of attention in the management of organizational life. However, it is necessary to be aware of the intertwined dimensions, generating different configurations of ethics practices in organizations to do this.

## Data Availability Statement

The original contributions presented in the study are included in the article/supplementary material, further inquiries can be directed to the corresponding author.

## Ethics Statement

Ethical review and approval was not required for the study on human participants in accordance with the local legislation and institutional requirements. The patients/participants provided their written informed consent to participate in this study.

## Author Contributions

SR, SP, and LG contributed to the conception and design of the study and organized the database. SP wrote the first draft of the manuscript. RZ and SD wrote sections of the manuscript. All authors contributed to manuscript revision, read, and approved the submitted version.

## Conflict of Interest

The authors declare that the research was conducted in the absence of any commercial or financial relationships that could be construed as a potential conflict of interest.

## Publisher’s Note

All claims expressed in this article are solely those of the authors and do not necessarily represent those of their affiliated organizations, or those of the publisher, the editors and the reviewers. Any product that may be evaluated in this article, or claim that may be made by its manufacturer, is not guaranteed or endorsed by the publisher.
